# On the electrocatalytical oxygen reduction reaction activity and stability of quaternary RhMo-doped PtNi/C octahedral nanocrystals†

**DOI:** 10.1039/d2sc01585d

**Published:** 2022-08-02

**Authors:** Elisabeth Hornberger, Malte Klingenhof, Shlomi Polani, Paul Paciok, Attila Kormányos, Raphaël Chattot, Katherine E. MacArthur, Xingli Wang, Lujin Pan, Jakub Drnec, Serhiy Cherevko, Marc Heggen, Rafal E. Dunin-Borkowski, Peter Strasser

**Affiliations:** Electrochemical Energy, Catalysis and Material Science Laboratory, Department of Chemistry, Technische Universität Berlin 10623 Berlin Germany polani@tu-berlin.de pstrasser@tu-berlin.de; Ernst Ruska-Centre for Microscopy and Spectroscopy with Electrons, Peter Grünberg Institute, Forschungszentrum Jülich GmbH 52425 Jülich Germany m.heggen@fz-juelich.de; Helmholtz-Institute Erlangen-Nürnberg for Renewable Energy (IEK-11), Forschungszentrum Jülich GmbH 91058 Erlangen Germany; ID 31 Beamline, BP 220, European Synchrotron Radiation Facility 38043 Grenoble France

## Abstract

Recently proposed bimetallic octahedral Pt–Ni electrocatalysts for the oxygen reduction reaction (ORR) in proton exchange membrane fuel cell (PEMFC) cathodes suffer from particle instabilities in the form of Ni corrosion and shape degradation. Advanced trimetallic Pt-based electrocatalysts have contributed to their catalytic performance and stability. In this work, we propose and analyse a novel quaternary octahedral (oh-)Pt nanoalloy concept with two distinct metals serving as stabilizing surface dopants. An efficient solvothermal one-pot strategy was developed for the preparation of shape-controlled oh-PtNi catalysts doped with Rh and Mo in its surface. The as-prepared quaternary octahedral PtNi(RhMo) catalysts showed exceptionally high ORR performance accompanied by improved activity and shape integrity after stability tests compared to previously reported bi- and tri-metallic systems. Synthesis, performance characteristics and degradation behaviour are investigated targeting deeper understanding for catalyst system improvement strategies. A number of different *operando* and on-line analysis techniques were employed to monitor the structural and elemental evolution, including identical location scanning transmission electron microscopy and energy dispersive X-ray analysis (IL-STEM-EDX), *operando* wide angle X-ray spectroscopy (WAXS), and on-line scanning flow cell inductively coupled plasma mass spectrometry (SFC-ICP-MS). Our studies show that doping PtNi octahedral catalysts with small amounts of Rh and Mo suppresses detrimental Pt diffusion and thus offers an attractive new family of shaped Pt alloy catalysts for deployment in PEMFC cathode layers.

## Abstract 2

PtNi nano-octahedra with Rh and Mo dopants are highly active catalysts for the oxygen reduction reaction with excellent stability and shape integrity. We investigate the morphological, structural, and compositional evolution during stability testing.

## Graphical abstract

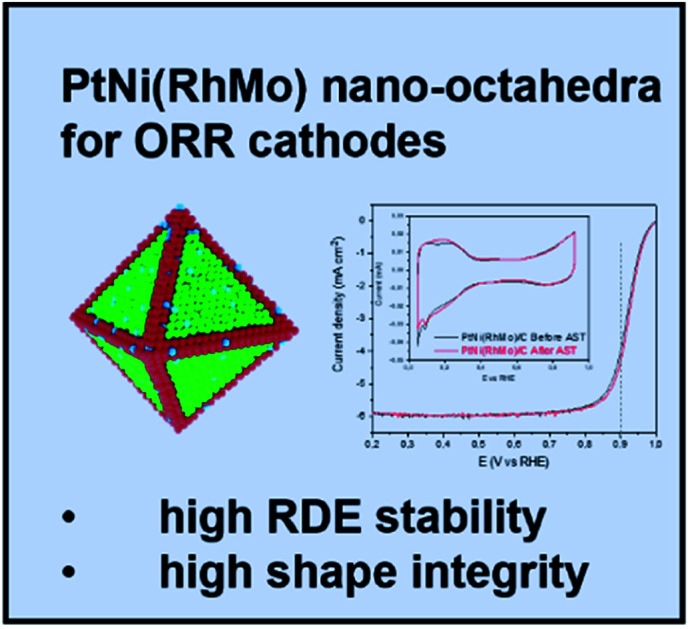

## Introduction

In the current climate and energy context, where the power generation and transportation sectors require breakthroughs to reduce anthropogenic carbon dioxide emissions, electrochemical conversion and storage devices such as proton exchange membrane fuel cells (PEMFCs) and water electrolysis (PEMWEs) represent appealing solutions. To date, the widespread commercialization of PEMFCs in stationary, portable, and hydrogen-powered fuel cell electric vehicles has been hampered by their still relatively high cost.^1^ Specifically in the transportation sector, medium- and heavy-duty vehicles are responsible for 24% of US greenhouse gas emission. To facilitate the decarbonization of those vehicles, durable catalyst materials for PEMFCs must be developed that fulfill the specific requirements for long-range and heavy load applications.^2,3^ As a result, scalable designs of new durable low Pt-based electrocatalysts for the oxygen reduction reaction (ORR) are essential.^4^

The incorporation of a 3d transition metal lowers the cost of a potential catalyst, while improving ORR kinetics by tailoring the d-band structure of Pt surfaces.^5^ Many Pt alloys with 3d transition metals such as Fe, Co, Ni, or Cu have been addressed in the literature.^6,7^ In addition to the composition, the surface structure of a catalyst is critical, as well, and must be carefully considered, because the ORR is a structure-sensitive reaction. In 2007, Stamenkovic *et al.* demonstrated the unique properties of annealed Pt_3_Ni (111) “Pt-skin” single crystal surfaces and their high ORR activity.^8^ In cubic crystal systems, nanoparticles with exclusive exposure of {111} sites have a tetrahedral or octahedral shape.

Since the first report by El-Sayed and his colleagues on the synthesis of cubic and tetrahedral Pt nanoparticles (NPs),^9^ there have been several publications addressing the physicochemical properties of shaped Pt-based nanoscale materials.^10^ Inspired by the exceptional ORR activity reported on an extended Pt skin (111) surface, as well as advances in the synthesis of shaped NPs, numerous studies have investigated methods to prepare octahedral (oh-)Pt_3_Ni NPs with well-defined (111) facets for the ORR.^11,12^

For example, Zou and Yang groups^13,14^ produced oh-Pt_3_Ni NPs based on surfactant directed reduction in organic solution using tungsten carbonyl or CO_(g)_ as the shaping agent. These particles showed a fourfold increase in specific activity (SA) compared to pure Pt/C electrocatalysts. Choi *et al.*^15^ reported high activity using a slightly modified method to prepare particles with less residual surfactant on the surface (*i.e.*, 17-fold higher mass activity and 51-fold higher SA). Carpenter *et al.*^16^ used dimethylformamide (DMF) as both the solvent and the reducing agent under solvothermal conditions and described an alternative surfactant-free technique for the generation of shaped Pt alloy NPs. The produced oh-PtNi NPs with a diameter of 12–15 nm had 6 times the mass activity (MA) of Pt/C and 10 times the SA of Pt/C. By modifying the Pt:Ni mixture over the reaction time, Cui^17^ and Gan^18^ were able to produce oh-PtNi catalysts with a diameter of about 9 nm and 10-fold ORR MA over Pt/C. Time resolved analysis of the formation mechanism revealed that the formation of Pt-rich hexapod-like nanocrystals was followed by Ni deposition in the 〈111〉 direction into the concave voids, resulting in oh-PtNi NPs with a Pt-rich hexapod core surrounded by Ni-rich (111) facets.^18^ Nevertheless, the performance of the oh-PtNi catalysts was an order of magnitude lower than that of the corresponding Pt_3_Ni (111) single crystal and they suffered from Ni leaching under electrochemical conditions, leading to concave octahedral corrosion patterns within the (111) facets and resulting in poor stability.^17,19^

Next, PtNi-based trimetallic oh-NPs were considered and proved a promising class of stable and active catalysts. The most studied dopants are Mo and Rh, both of which promote the segregation of Pt on the surface.^20,21^ Huang *et al.*^22^ fabricated Mo-doped oh-PtNi NPs with a remarkable ORR of 6.98 A mg_Pt_^−1^ and only a 5% decrease in activity after 8000 potential cycles. Dionigi *et al.*^23^ later reported the realization of exceptionally high catalytic oxygen electroreduction activities of Mo-doped oh-PtNi catalysts from conventional thin-film rotating disk electrode screenings (3.43 ± 0.35 A mg_Pt_^−1^ at 0.9 V_RHE_) to a membrane electrode assembly (MEA)-based single fuel cell test with a sustained Pt MA of 0.45 A mg_Pt_^−1^ at 0.9 V_cell_, one of the highest performances ever reported for advanced shaped Pt alloys in real devices. Scanning transmission electron microscopy with energy dispersive X-ray analysis (STEM-EDX) revealed that Mo predominantly occupies the Pt-rich edges and tips of the elementally anisotropic oh-PtNi NPs. The study demonstrates at the atomic level how Mo surface atoms influence the Ni surface composition, which in turn leads to the exceptionally high experimental catalytic ORR reactivity.

Beermann *et al.*^24^ reported the unusual behavior of Rh-doped oh-PtNi NPs with high activities up to 1.14 A mg_Pt_^−1^ and improved performance and shape integrity over previous bimetallic oh-PtNi particles. The synthesis, electrocatalytic performance of the particles towards the ORR and atomic degradation mechanisms were investigated with emphasis on better understanding of morphological degradation and stability. The NPs largely maintained their octahedral shape after 30 000 potential cycles, while undoped reference bimetallic NPs lost their octahedral shape after only 8000 cycles in the same potential window. The results suggest that the migration of Pt surface atoms, rather than Ni dissolution, was the main cause of the loss of the octahedral shape in the PtNi NPs. The synergistic effects of two different surface dopants on the surface of PtNi nanooctahedra have remained unexplored to date.

Herein, we present a synthesis–structure–activity study of previously unexplored Ni-rich quaternary Mo- and Rh-doped oh-PtNi NP electrocatalysts (hereafter oh-PtNi(RhMo)). This family of catalysts showed unusually high ORR performance and electrochemical stability compared to a Ni-rich oh-PtNi catalyst and offers benefits compared to its ternary peers. Also, the quaternary alloy materials displayed favorable retention of activity after an accelerated stress test (AST). The catalyst was synthesized using a modified solvothermal method, in which the Rh precursor was added according to a synthesis protocol previously published by the Strasser group for Mo-doped oh-PtNi catalysts.^25^ We show that Rh has a positive effect on the performance and durability of quaternary oh-PtNi(RhMo) catalysts representing a new class of active and stable catalysts for the ORR.

## Results and discussion

**Fig. 1:**
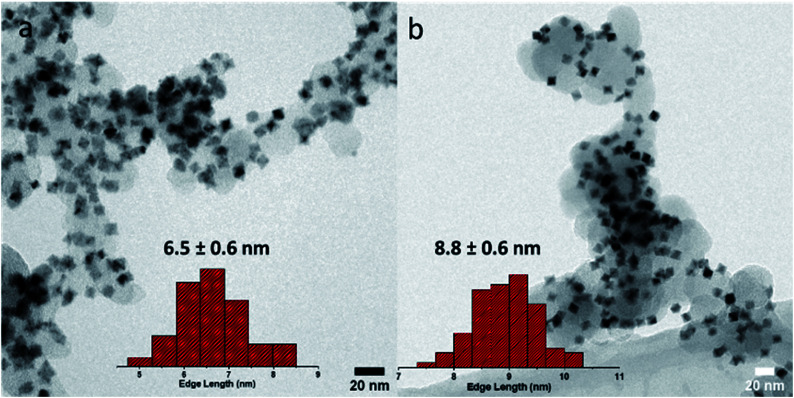
TEM micrographs of (a) binary oh-PtNi and (b) oh-PtNi(RhMo); the insets show the corresponding mean edge-length size distribution histogram.

Oh-PtNi(RhMo) NPs containing 1.4 and 0.4 atomic% Rh and Mo, respectively, were synthesized by a solvothermal method using benzyl alcohol as the solvent and reducing agent in a sealed pressure flask. For comparison and reference, bimetallic oh-PtNi particles were synthesized by the same protocol without dopant precursors. We note that ternary PtNi(Rh) and alike reference catalysts were not accessible using this solvothermal route (Fig. S1†). Full details of the experimental methods and syntheses can be found in the ESI.†Fig. 1 shows the transmission electron microscopy (TEM) images of the binary and quaternary catalysts, confirming a uniform distribution of the octahedral NPs onto the carbon support. The NPs show an excellent shape uniformity.

**Fig. 2:**
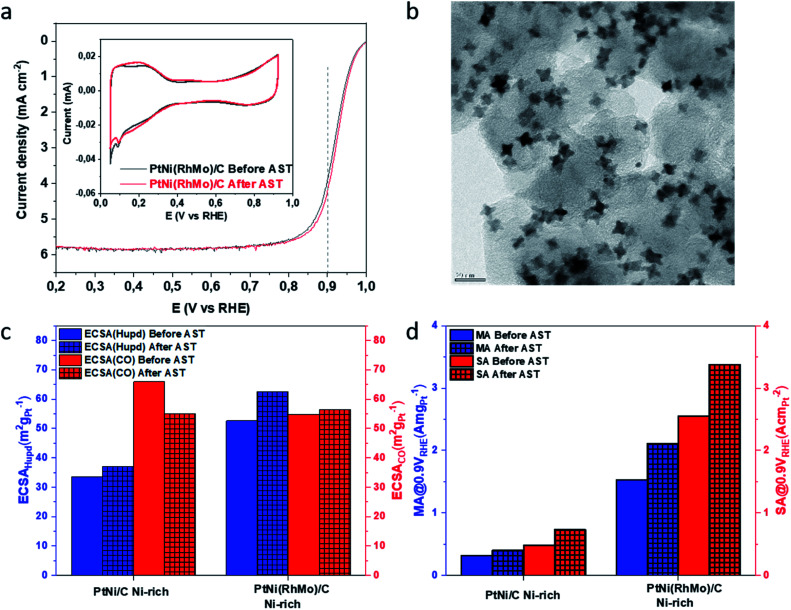
Electrochemical and morphological characterization of oh-PtNi(RhMo) and oh-PtNi. (a) Linear sweep voltammetry curves before and after the AST (10 800 cycles between 0.6 and 0.95 V_RHE_ at a scan rate 1 V s^−1^). Inset shows the cyclic voltammetry profile before and after the AST. (b) TEM micrograph of the catalyst material after the AST as probed directly from the RDE working electrode. (c) ECSA values derived from H_upd_ and CO before and after the AST. (d) MA and SA values evaluated at 0.9 V_RHE_ before and after the AST.

ICP-OES analysis shows that the Ni content of the doped NPs is slightly lower than that of the binary NPs (see Table S1 in the ESI†). Typically, the reaction temperature is above the boiling point of acetylacetonate (acac, 153 °C and 140 °C, respectively), which saturates the gas phase as the reaction proceeds. When Mo(CO)_6_ precursors are added to the reaction, the system becomes more complex. The presence of CO ligands has a strong effect on the particle size and Ni content. CO(g) increases the gas phase pressure at the beginning of the synthesis, leading to a slower reduction of Pt^II^ to Pt^0^. Since the higher gas phase pressure counteracts the transition of acac(g) into the gas phase, the reaction equilibrium shifts to the side of the precursors. This results in a bigger average edge length and a lower Ni content for the PtNi(RhMo) NPs compared to the reaction equilibrium without CO ligands. The electrocatalysts were investigated using the rotating disk electrode (RDE) technique in acidic media (0.1 M HClO_4_). The alloy nanocatalysts were applied as a thin catalyst film to the RDE in a non-preleached format. Fig. 2a shows the ORR activity curves and cyclic voltammetry (CV) shape evaluation for oh-PtNi(RhMo) after activation (50 cycles, 0.05–0.95 V_RHE_, 100 mV s^−1^, and N_2_-saturated electrolyte) and after an accelerated stress test (AST) (10 800 cycles, 0.6–0.95 V_RHE_, 1 V s^−1^, and N_2_-saturated electrolyte). The ORR curves show a stable diffusion-limited current region and a shift of the mixed (kinetic and mass transport) current region towards higher potentials after the AST, as suggested for Rh-doped catalysts.^24^ The current response features in the CV profile at low potentials in the hydrogen under potential deposition (H_upd_) region of oh-PtNi(RhMo) increase slightly, indicating alteration of the surface structure and faceting after conducting the AST, probably due to an increased contribution of (110).^8^ The CV profile of oh-PtNi started with less pronounced features in the H_upd_ region, which changed slightly after the AST (Fig. S2a†). Fig. 2b and S2b† show the TEM images of the oh-PtNi(RhMo) and oh-PtNi catalyst layers, respectively, after the electrochemical tests, deposited directly from the RDE working electrode on a TEM grid. The oh-PtNi(RhMo) particles exhibit a pronounced concavity after the AST, which could contribute to the development of the H_upd_ profile corresponding to the exposed facets of concaved NP, while oh-PtNi particles show a quasi-spherical shape. Fig. S3† shows additional STEM-EDX analysis of the oh-PtNi(RhMo) particles after the electrochemical tests. The high-angle annular dark-field (HAADF) STEM image shows uniformly distributed oh-PtNi(RhMo) particles on the carbon support. The oh-PtNi(RhMo) particles were examined in detail by EDX elemental mapping. After the RDE test, the distribution of the elements Pt, Ni and Rh is homogeneous throughout the particle. Adsorbates on the particle surface, in particular oxygen during the determination of the ORR activity and CO to estimate the electrochemically active surface area (ECSA_CO_), can induce surface segregation processes.^17,26,27^ Fig. S4† shows a HAADF-STEM image of the binary oh-PtNi particles after electrochemical testing. The shape of the oh-PtNi particles transforms into a more rounded spherical shape during the AST and the particles show a concave shape unlike oh-PtNi(RhMo). Complementary EDX elemental mapping shows the formation of a distinct Pt-rich shell.

To further characterize the electrochemical stability of the catalysts, key parameters were evaluated and are summarized in Fig. 2c and d and compared to relevant literature in Table S2.† The ECSA_H_upd__ for the quaternary oh-PtNi(RhMo) was extracted at 52.6 m^2^ g_Pt_^−1^ and 69% larger than that of undoped oh-PtNi. These values can be compared with the values of ternary doped oh-PtNi(M) catalysts from the literature for oh-PtNi(Rh)^24^ of 31 m^2^ g_Pt_^−1^ and oh-PtNi(Mo)^23^ of 38 m^2^ g_Pt_^−1^ and oh-PtNi(Mo)^25^ of 55.0 m^2^ g_Pt_^−1^. The ECSA_H_upd__ of oh-PtNi(RhMo) and oh-PtNi increased to 62.6 and 37.1 m^2^ g_Pt_^−1^, respectively, after ASTs. A similar trend and values have been observed for oh-PtNi(Mo).^25^ Complementary to the ECSA_H_upd__, CO electrooxidation experiments were performed to take advantage of the full ECSA analysis for accurate SA evaluation. CO-stripping was performed to evaluate the ECSAs_CO_ (Fig. S5†). Both H_upd_ and CO are very surface-sensitive species. The alteration of the electronic structure of the catalytically active surface of the NPs can result in weakened or strengthen Pt-adsorbate interactions. The ratio between *Q*_CO_ and *Q*_H_upd__ can give more detailed insights. Van der Vliet *et al.* related these values to ∼1 for pure Pt (“skeleton-type”) surfaces and ∼1.5 for thermally annealed Pt “skin-type” surfaces.^28^ oh-PtNi(RhMo) shows compared to the ECSA_H_upd__ only a slightly higher ECSA_CO_ of 54.8 m^2^g_Pt_^−1^. A similar trend and values were also observed for oh-PtNi(Mo).^25^ The *Q*_CO_/*Q*_H_upd__ ratio for oh-PtNi(RhMo) is 1.01. This value is close to that of ∼1 for Pt-pure oh-Pt/C.^29^ Surprisingly, the ECSA_CO_ for oh-PtNi compared to the ECSA_H_upd__ is almost twice as high at 65.9 m^2^g_Pt_^−1^ resulting in a *Q*_CO_/*Q*_H_upd__ ratio of 1.71. Rudi *et al.* concluded that values ≥1.5 are not necessarily a reasonable characteristic feature for Pt “skin-type” surfaces.^26^ To investigate the origin of this behaviour for the presented Ni-rich oh-PtNi sample, more detailed characterization techniques are performed and discussed in the following sections.

Interestingly, the ECSA_CO_ values decrease slightly for oh-PtNi and oh-PtNi(Mo)^25^ and remain similar within the error bar for oh-PtNi(RhMo) after the ASTs, which could be interpreted as an indication of small Ni losses due to leaching processes that slightly lower the ECSA_CO_ but increase the ECSA_H_upd__ due to the roughening of the particle surface. The Pt mass-based ORR activity (MA) was determined at a scan rate of 20 mV s^−1^. oh-Pt_18_Ni_82_ shows a MA of 0.32 A mg_Pt_^−1^, which is comparable to an optimized spherical pure Pt/C catalyst with a MA of 0.31 A mg_Pt_^−1^ and pure oh-Pt/C with a MA of 0.36 A mg_Pt_^−1^. Due to the high Ni ratio in this binary catalyst a careful literature comparison is necessary. Moderately Ni-rich oh-Pt_40_Ni_60_ showed more than twice the MA of oh-Pt_18_Ni_82_.^17,29^ The MA was dramatically increased with Mo and Rh doping for oh-PtNi(RhMo) to 1.53 A mg_Pt_^−1^, which is higher than that of oh-PtNi(Rh) with 0.82 A mg_Pt_^−1^ of Beermann *et al.* but lower than that of oh-PtNi(Mo) with 2.27, 3.43 and 6.98 A mg_Pt_^−1^ of Polani *et al.*, Dionigi *et al.* and Huang *et al.* respectively. We note that the exceptionally high value of 6.98 A mg_Pt_^−1^ has never been independently reproduced. In addition, a binary oh-Pt_33_Ni_67_ catalyst was shown to have a MA value of 1.7 A mg_Pt_^−1^, which was decreased to 1.44 A mg_Pt_^−1^ after 4000 cycles of the AST (see Table S2†).^30^ In our study, both catalysts show increased MA after the AST. This is in contrast to oh-PtNi(Mo)^25^ that showed a 30% decreased MA after the AST. A measure of intrinsic activity is accessible by normalizing the Pt mass-based ORR activity (A mg_Pt_^−1^) and the Pt mass-based ECSA (m^2^ g_Pt_^−1^). This parameter describes the specific, Pt-mass free, real-surface-based ORR activity (SA). oh-PtNi(RhMo) shows an almost ∼4.8-fold increased SA compared to oh-PtNi and 67% of the oh-PtNi(Mo)^25^ counterpart initial SA. After ASTs, oh-PtNi(RhMo) and oh-PtNi show an increased SA_CO_ of 47% and 42%, respectively, while oh-PtNi(Mo)^25^ showed a decreased SA_CO_ of 21%. By comparing the behavior of the Mo-doped catalyst with that of the Mo- and Rh-doped catalyst, a positive stability effect of Rh doping is evident. The increase in activity after the AST for oh-PtNi(RhMo) could be due to the presence of oxidized Mo and Rh atoms, which stabilize sites with lower coordination and lead to the formation of a concave octahedron by Ni leaching from the facets.^20^ The geometry and adsorption energy of oxygen species for a surface strongly correlate with the ORR activity of Pt sites, namely that concave defects (with a generalized coordination number 
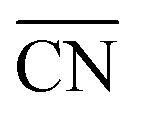
 > 7.5) exhibit enhanced activity and can even outperform pristine Pt(111) sites. Thus, oh-PtNi(RhMo) shows promising ORR activity and stability properties for a feasible electrocatalyst. The origin of increased activity after the AST for oh-PtNi will be discussed in the following section.

**Fig. 3:**
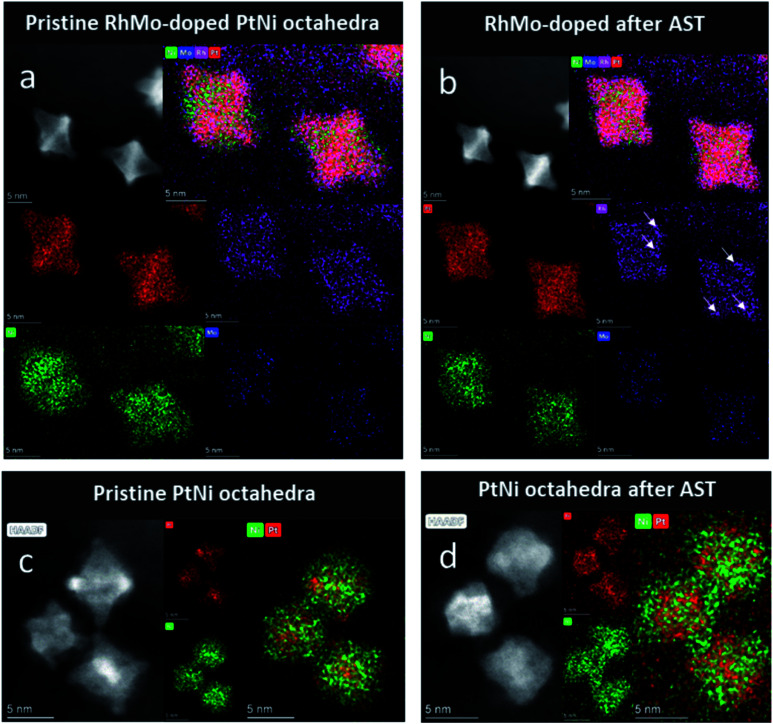
IL-HAADF-STEM images (grey) and STEM-EDX composition maps of the pristine state (a) and (c) and after an AST of 10 800 cycles between 0.6 and 0.95 V_RHE_ at a scan rate 1 V s^−1^ (b) and (d) for RhMo-doped PtNi (Pt in red, Ni in green, Rh in purple, and Mo in blue) and PtNi octahedra.

Identical location high-angle annular dark field (IL-HAADF)-STEM and IL-STEM-EDX (Fig. 3 and S6–S9†) allow the direct visual tracking of structural and morphological changes in the catalyst due to dissolution/redeposition of different metals, migration/aggregation of the Pt-based NPs and corrosion of the carbon support.^31–33^ IL-STEM was performed on oh-PtNi(RhMo) and oh-PtNi catalysts applying only the ASTs in a N_2_-saturated electrolyte (no pre- and post-ORR activity measurement in a O_2_-saturated electrolyte was performed). The oh-PtNi(RhMo) catalyst can be described as an elemental composite of a Pt-rich hexapod that determines the overall size of the octahedral particle and Ni mainly forms Ni rich facets. The elemental localization of Rh and Mo is limited due to the weak and noisy EDX signal for both elements; however a preferential distribution on edges and vertices can be observed (see arrows in Fig. 3). Due to the lower degree of Mo doping, the elemental localization of Mo is limited. After the AST, the overall morphology of the octahedral shape of PtNi(RhMo) remains intact. The elemental distribution of Pt is preserved in the Pt-rich hexapod. Ni on the facets leaches off to a small extent accompanied by Rh and Mo, resulting in some concavity of the octahedral particles. The evolution of the metallic at% derived from several replications using IL-STEM-EDX is shown in Table S3.† Overall, the PtNi(RhMo) particles show exceptional morphological stability and elemental integrity after the AST. In addition to the less pronounced octahedral morphology, the oh-PtNi catalyst shows a comparable Pt-rich hexapod and Ni-rich facets to oh-PtNi(RhMo). However, careful examination of the Ni elemental distribution maps suggests the presence of a Ni shell around the oh-PtNi particles. The Ni shell is accompanied by a congruent O shell, suggesting the formation of an acid-stable NiO_*x*_ shell (Fig. S9†). This confirms the hypothesis of accumulated (and electrochemically active) Ni species on the particle surface when comparing the drastic difference between ECSA_H_upd__ and ECSA_CO_ before the AST, which was evaluated by RDE characterization. After the AST, the oh-PtNi particles widely lost their octahedral shapes. The Pt-rich hexapod and Ni-rich facets became less defined and led to a higher degree of alloy formation, while larger amounts of Ni are leached. The O shell seems to become more permeable, which could account for the observed increase in both SA and MA (Fig. S9†). The evidence of Ni leaching in smaller amounts in oh-PtNi(RhMo) and in larger amounts in oh-PtNi is also in accordance with the proposed origin for the evolution of both ECSA_H_upd__ and ECSA_CO_.

Theoretical calculations by Cao and Mueller^20^ have shown that in binary oh-Pt_3_Ni NPs under oxidizing conditions, both the Pt and Ni atoms are most sensitive to dissolution at the vertices, which can lead to the loss of the octahedral shape. On the other hand, in Mo-doped Pt_3_Ni NPs, the Pt and Ni atoms at the facets are most susceptible to dissolution from the surface because the formation of Mo oxide at the vertices and edges provides additional stabilization to the Pt/Ni atoms at the vertices. Consequently, the edges (including the vertices) of the oh-Pt_3_Ni NPs are the ones that are further stabilized by Mo doping, which is consistent with the experimental observation of the strongly preserved octahedral shape of the oh-PtNi(RhMo) catalysts.

**Fig. 4:**
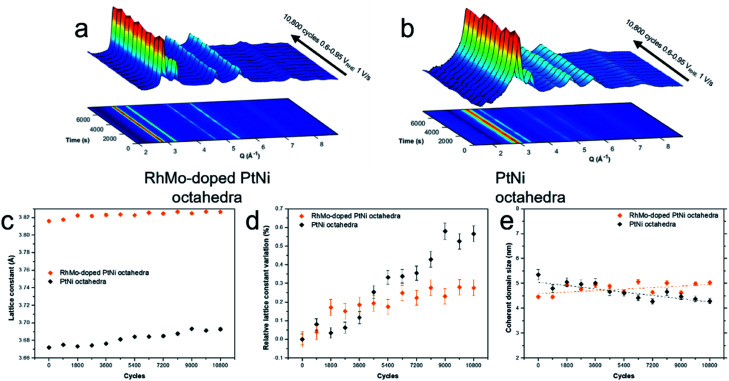
3D representation and corresponding 2D projection of WAXS pattern intensities plotted as a function of time and the momentum transfer *Q* recorded in the AST (10,800 cycles between 0.6 and 0.95 V_RHE_ at a scan rate 1 V s^−1^) for (a) oh-PtNi(RhMo) octahedra and (b) PtNi octahedra. Results from Rietveld refinement analysis in terms the evolution of (c) the lattice constant, (d) relative lattice constant variation and (e) coherent domain size.

To further elucidate the structural composition of the oh-PtNi(RhMo) and oh-PtNi catalysts, wide angle X-ray scattering (WAXS) experiments were performed *operando* at beamline ID31 of the European Synchrotron Radiation Facility (ESRF). Fig. 4 shows the diffraction data obtained. The catalysts were deposited as thin films on a GC substrate that acted as a working electrode and investigated in a grazing incident electrochemical cell using liquid acidic media. The analysis of *operando* WAXS measurements on extremely thin films supported on a GC is extremely sensitive to the presence of GC reflections in the diffraction patterns. This particularly affects the PtNi [111] reflection, which contributes most to the overall pattern. Therefore, partial Rietveld refinements of the WAXS patterns were performed within the region without GC reflections. Fig. S10† shows exemplary fits of the WAXS partial patterns subtracted from the background. For oh-PtNi(RhMo), a single (face-centred cubic) fcc-PtM phase was sufficient, while oh-PtNi was best fitted with a single fcc-PtM phase and an fcc-NiO phase. This confirms the finding of a NiO_*x*_ shell for oh-PtNi from the studies of IL-STEM-EDX. Using Rietveld refinement analysis, the lattice constant was extracted (Fig. 4c). The initial value of the lattice constant for oh-PtNi(RhMo) is larger than for oh-PtNi, which can be rationalized by the difference in the elemental composition of oh-PtNi(RhMo) and Ni-richer oh-PtNi. Both catalysts show lattice expansion during the AST. This can be associated with surface Ni leaching resulting in Pt-richer surfaces. A direct comparison of the evolution of the relative lattice parameters for both catalysts reveals the significant advantages of quaternary doping with Rh and Mo in terms of non-precious metal retention (Fig. 4d). While oh-PtNi showed a continuous lattice expansion, oh-PtNi(RhMo) reached a nearly steady state after ∼1800 cycles. The size of the coherent domains during the potential cycles follows opposite trends. While the coherent domain size of oh-PtNi decreased, oh-PtNi(RhMo) showed increasing values. This indicates that oh-PtNi(RhMo) undergoes very mild Ostwald ripening, but retains its structural integrity over test time. In contrast, the binary NPs initially had 82 Ni at%, which was higher than that of the quaternary (66 at%), leading to more severe Ni dissolution and loss of structural integrity, while the Ostwald ripening is a relatively minor process. This is demonstrated in our measurement as a decrease of coherent domain size. Overall, the results of the *operando* WAXS experiments fully support the hypothesis that oh-PtNi(RhMo) exhibits superior stability characteristics in terms of both structural stability and prevention of Ni dissolution.

To gain a more detailed understanding of the dissolution behavior, particularly of Ni, on-line inductively coupled plasma mass spectrometry (on-line ICP-MS) experiments were conducted.^34–36^ Fig. S11 and S12† show the dissolution profiles recorded for the oh-PtNi and oh-PtNi(RhMo) samples. On top of the AST protocol (10 800 scans with 1 V s^−1^ in between 0.6 and 0.95 V_RHE_), an additional CV was recorded in a wide potential window to determine the onset of dissolution and to glean insights into the stability of the catalysts in a wider potential window. Three dissolution features can be distinguished by looking at the dissolution profiles, except for Mo for which only one dissolution peak can be identified. The first and most pronounced peak (except for Pt) corresponds to the dissolution that occurred when the cell came in contact with the catalyst spot. This peak rapidly decreased until the end of the galvanostatic hold (five minutes applying *j* = 0 mA cm^−2^ current density). The second peak emerged when the potential was shifted from open circuit potential (OCP) to the starting potential of the AST protocol (0.6 V_RHE_). Apart from these, no metal dissolution was detected during the AST protocol. Finally, increased dissolution was observed when the potential was scanned towards significantly more positive values reaching 1.5 V_RHE_. The only exception is Mo: here, metal dissolution was observed only during contacting. Two dissolution peaks emerged for Pt and Rh during the final CV corresponding to the formation of PtO_*x*_ and RhO_*x*_ (possibly Rh_2_O, RhO, and Rh_2_O_3_)^37^ and their subsequent reduction during the reverse scan. The characteristics of the dissolution features match well with literature data for both Pt and Rh.^38,39^ On the other hand, only one dissolution peak was detected for Ni.

**Fig. 5:**
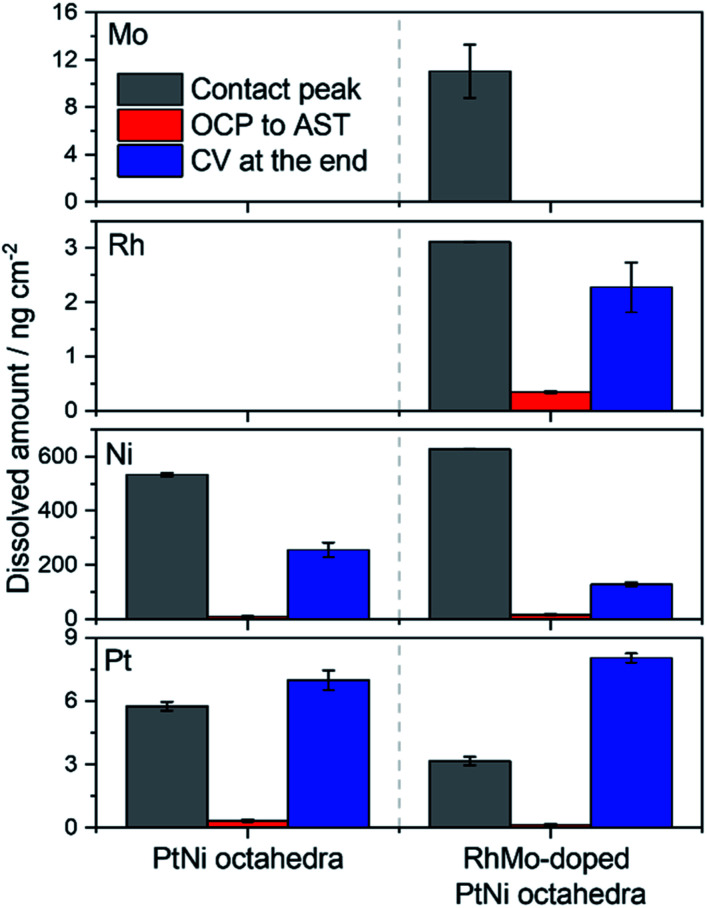
Dissolved amounts calculated by integrating the dissolution profiles of oh-PtNi and oh-PtNi(RhMo) presented in Fig. S11 and S12.†

The dissolution of Pt, Ni and Rh started at 1.10, 1.01, and 1.01 V_RHE_, respectively. Since Mo dissolution was not detected during the final CV, the onset potential of dissolution was not determined. While Pt dissolution starts at an expected potential, Ni and Rh dissolution commence above the values expected from thermodynamics.^37^ Based on these observations, it can be concluded that Pt controls the dissolution of both metals at the end of the AST protocol. This means that all Ni atoms present on the surface of the electrocatalysts are immediately passivated/dissolved when the cell comes in contact with the drop-cast catalyst spot and Ni dissolution is only possible when Pt starts to dissolve making fresh Ni sites available. A similar phenomenon was observed for Mo-doped oh-PtNi samples earlier.^25,35^ The amount of Rh is very small in the oh-PtNi(RhMo) sample (1.4 at% based on the ICP-OES measurement presented in Table S1†). According to the IL-HAADF-STEM images presented in Fig. 3, Rh atoms are evenly scattered both on the facets and edges of the hexapods and no Rh enriched regions can be identified. Thus, Rh dissolution is also controlled by the main constituents of the alloy, namely, Pt and Ni. The Mo content of the alloy is even smaller (0.4 at%), and hence all surface Mo is either dissolved or oxidized (passivated) upon contacting leading to no Mo dissolution during the final CV in the extended potential window. To glean quantitative insights, all dissolution features were integrated. Fig. 5 shows the resulting dissolved amounts for both the oh-PtNi and oh-PtNi(RhMo) samples (to better visualize the dissolved amounts when the potential was shifted from OCP to 0.6 V_RHE_; sections of the figure were magnified and can be seen in Fig. S13†). Since both alloy samples are Ni-rich and Ni is not stable in the acidic environment, the dissolved amount of Ni was the highest among the four investigated metals. Interestingly, Ni dissolution during contacting is relatively similar for the pristine and doped samples, despite the lower Ni content of the latter (82 at% *vs.* 65.6 at%), while the order is reversed for the CV recorded in the extended potential window. This suggests that the initial Ni content on the electrocatalyst surface was higher for the RhMo-doped sample at the beginning of the experiment. This was confirmed by the IL-STEM-EDX and WAXS measurements (Table S3† and Fig. 4) showing the formation of a NiO_*x*_ shell on the surface of the oh-PtNi sample (resulting in a lower surface Ni content). The total amount of dissolved Ni is in the ballpark of what was reported in precedent studies.^40^ The dissolved amount of the other three metals is at least two orders of magnitude smaller. The amount of dissolved Rh is the smallest, which is followed by Pt. Pt dissolution during contacting is around 50% less for the doped sample, while it is similar for both catalysts during the final CV. The amount of dissolved Mo is approximately similar to the amount of Pt dissolved during the whole protocol, despite the very low Mo-content of the RhMo doped sample. Finally, the post-AST composition of both samples was calculated (Table S3†). Similarly to the IL-STEM-EDX measurements, the Pt : Ni ratio notably increased in both the oh-PtNi and oh-PtNi(RhMo) samples along with the increase of the Rh-content and decrease of the Mo-content.

Compared to the initial total metal loading, approximately 10.5% of the metal loading of the PtNi octahedra sample was lost during the AST protocol. The total metal loading lost due to dissolution is notably higher (14.8%) for the RhMo-doped PtNi octahedra sample thanks to the ≈15% lower initial total metal loading of this sample, to the higher Ni dissolution rate and the additional dissolution of Mo and Rh. We note that most of the lost metal mass is Ni (two magnitudes higher than all the other metals) and that most metals were dissolved when the cell came in contact with the catalyst thin films. Unfortunately, the presence of NiO_*x*_ on the surface makes the comparability of the on-line ICP-MS data recorded for oh-PtNi to the IL-STEM data impossible (all post-AST compositions are based on the ICP-OES data). On the other hand, the post-AST composition of the PtNi(RhMo) samples is comparable. The Pt-content of the post-AST sample is about 15 at% lower than the value resulted in the IL-STEM-EDX measurements (59 *vs.* 43.88). The same goes for the Ni content, but the difference is more evident (54.16 *vs.* 28 at%). In contrast to the on-line ICP-MS data, both the Rh- (5.4 *vs.* 1.78 at%), and Mo-content (7.6 *vs.* 0.18 at%) increased in the post-AST samples according to the IL-STEM-EDX data. The reason behind this difference might be two-fold: (i) pre-AST IL-STEM EDX composition already showed notable differences compared to the composition determined by ICP-OES (lower Ni content in parallel with higher Rh and Mo contents) and (ii) IL-STEM-EDX data were recorded only for a few particles, while the on-line ICP-MS post-AST composition was calculated for the bulk material. The higher Mo content measured with IL-STEM-EDX might also explain the unexpectedly high Mo dissolution experienced for the oh-PtNi(RhMo) sample.

## Conclusion

This study has addressed the synthesis, structure, and ORR electrocatalysis of novel quaternary octahedrally shaped oh-PtNiRhMo nanoalloys, with particular focus on compositional and structural stability benefits. To achieve this, we have developed a synthetic solvothermal route for quaternary Rh- and Mo-doped oh-PtNi catalysts with well-defined octahedral shape. The resulting Ni-rich oh-PtNi(RhMo) particles were tested for the electrochemical ORR and showed exceptional and improved activity and stability. By adding dopant precursors alone, we were able to suppress the formation of a NiO_*x*_ shell on the oh-PtNi(RhMo) particle surface and thus improve the electrochemical performance compared to their bimetallic counterpart. After the stability test, the activity was improved, and the octahedral shape remained almost unchanged. In contrast, the reference particles of oh-PtNi showed shape degradation after the AST. These observations are explained by detailed microstructural studies of the atomic rearrangement processes on the surfaces of the two catalysts using the complementary advanced techniques of IL-STEM-EDX, *operando* WAXS and on-line SFC-ICP-MS techniques. It was found that main leaching events from both catalysts occurred when the working electrode encounters the electrolyte and the potential changes from OCP to AST conditions. The presence of Rh and Mo enhances structural integrity by suppressing the migration of Pt surface atoms. These results can serve as a guide for a new class of highly active and exceptionally stable shaped electrocatalysts that can be incorporated into the cathode of PEMFCs.

## Author contributions

The manuscript was written through contributions of all authors.

## Conflicts of interest

There are no conflicts to declare.

## Supplementary Material

SC-013-D2SC01585D-s001
