# Supplementary material for: On the electrocatalytical oxygen reduction reaction activity and stability of quaternary RhMo-doped PtNi/C octahedral nanocrystals
Source: Chem Sci. 2022 Aug 2;13(32):9295–304. doi: 10.1039/d2sc01585d (PMC9384817; doi:10.1039/d2sc01585d)
Supplement: SC-013-D2SC01585D-s001 [file SC-013-D2SC01585D-s001.pdf]

Electronic Supplementary Material (ESI) for Chemical Science.
This journal is © The Royal Society of Chemistry 2022

Supporting Information

On the Electrocatalytical Oxygen Reduction Reaction Activity and Stability of Quaternary RhMo-doped PtNi/C Octahedral Nanocrystals

Elisabeth Hornberger, Malte Klingenhof, Shlomi Polani,* Paul Paciok, Attila Kormányos, Raphaël Chattot, Katherine E. MacArthur, Xingli Wang, Lujin Pan, Jakub Drnec, Serhiy Cherevko, Marc Heggen,* Rafal E. Dunin-Borkowski, and Peter Strasser*

Table of Contents

Experimental Procedures (page 2)

Results and Discussion (page 5)

References (page 18)

Author Contributions (page 18)

Experimental Procedures

Materials.
Platinum (II) acetylacetonate ($Pt(acac)_2$, ≥98%) was obtained from Acros, nickel (II) acetylacetonate ($Ni(acac)_2$, ≥98%), rhodium acetylacetonate ($Rh(acac)_3$, 97.0%), molybdenum hexacarbonyl ($Mo(CO)_6$, 99.9%), and Nafion solution (5% w/w) were obtained from Sigma Aldrich. Platinum on activated carbon powder, from Johnson Matthey Fuel Cells (Pt/C, 50 Ptw%). Dicarbonyl(acetylacetonato)rhodium ($Rh(acac)(CO)_2$, 97%), polyvinylpyrrolidone (M. W. 10,000) and benzoic acid (99%) were obtained from Alfa Aesar. Benzyl alcohol (≥99%) was purchased from Carl-Roth, acetone and ethanol were purchased from VWR International. Carbon black (Vulcan XC -72) was obtained from Cabot Corporation. All chemicals were used as received.

Synthesis of PtNi/C octahedral NPs.
Synthesis of PtNi/C Ni-rich with 6 nm edge length, PtNi: 0.64 mg $Pt(acac)_2$, 200 mg $Ni(acac)_2$, 0.64 g PVP (10k) and 0.4 g benzoic acid in 40 mL benzyl alcohol (BA). The 100 mL pressure glass flask was then stoppered and stirred vigorously for 1 h at RT. Then the flask was heated to 150 ℃ with a ramp of 5 ℃ min^{-1}. After 12 h of reaction, the reaction solution was allowed to cool to RT, then 100 mg of carbon (XC 72R) in 10 ml of BA was added to the reaction solution after stirring overnight. After stirring overnight, the solution was purified with a mixture of ethanol and acetone using a centrifuge.

Synthesis of PtNi(RhMo)/C octahedral NPs.
In a typical synthesis of PtNi(RhMo)/C Ni-rich with 9 nm edge length: 0.64 mg $Pt(acac)_2$, 200 mg $Ni(acac)_2$, 20 mg $Mo(CO)_6$, 20 mg $Rh(acac)_3$, 0.64 g PVP (10k) and 0.4 g benzoic acid in 40 mL BA. The 100 mL pressure glass flask was then stoppered and placed in a heating mantle for 1 h with vigorous stirring at 60 ℃. Then the flask was heated to 150 ℃ with a ramp of 5 ℃ min^{-1}. After 12 h of reaction, the reaction solution was allowed to cool to RT and then 100 mg of carbon (XC 72R) in 10 ml of BA was added to the reaction solution after stirring overnight. After stirring overnight, the solution was purified with a mixture of ethanol and acetone using a centrifuge.

Synthesis of PtNi(Rh)/C NPs.
In a typical synthesis of PtNi(Rh)/C Ni-rich: 0.64 mg $Pt(acac)_2$, 200 mg $Ni(acac)_2$, 20 mg $Rh(acac)_3$, 0.64 g PVP (10k) and 0.4 g benzoic acid in 40 mL BA. The 100 mL pressure glass flask was then stoppered and placed in a heating mantle for 1 h with vigorous stirring at 60 ℃. Then the flask was heated to 150 ℃ with a ramp of 5 ℃ min^{-1}. After 12 h of reaction, the reaction solution was allowed to cool to RT, then the solution was purified with a mixture of ethanol and acetone using a centrifuge. We also tried to increase $Rh(acac)_3$ amount to 40 mg and use different Rh precursor like $Rh(acac)(CO)_2$ 13 mg.

Morphological and Structural and Elemental Characterization.
Transmission electron microscopy (TEM). Micrographs were acquired using a FEI Tecnai G2 20 S- TWIN with a LaB_6 cathode at an accelerating voltage of 200 kV and a resolution limit of 0.24 nm. Samples were dispersed in ethanol and dropped onto a Cu grid.
High resolution (HR)TEM and scanning TEM-energy dispersive spectroscopy (STEM-EDS). The high-resolution EDS elemental maps were acquired on a Thermo-Fisher probe corrected Titan 80-20 electron microscope fitted with a four quadrant Super-X EDX detector. The experiments were carried out at an operating at a voltage of 80 kV, to minimize particle damage and maximize X-ray counts, with a probe convergence angle of 25mrad. EDX quantification was carried out using the partial ionization cross section approach.
Identical location (IL) -STEM measurements. 10µl of an aqueous catalyst suspension (0.05gcat l^{-1}) was drop-casted on a gold TEM finder grid (S147A9, Plano GmbH) and dried. After acquiring several high angle annular dark field STEM images of different regions and the corresponding elemental maps, the catalyst-coated TEM grid was used as a working electrode for the AST consisting of 10,800 cycles between 0.6 and 0.95 V_{RHE} at 1V s^{-1} in Ar-saturated 0.1 M $HClO_4$ at RT. A Gaskatel HydroFlex RHE was used as the reference electrode and a graphite rod as the counter electrode. The potential was controlled with a Bio-Logic Science Instruments SP-50 potentiostat. The ohmic potential drop (iR drop) was measured and corrected using the current interruption method. After AST, the grid was rinsed with ultrapure water to remove electrolyte residues and dried. Subsequently, the grid was analyzed again at identical locations.
Inductively coupled plasma - optical emission spectrometry (ICP-OES) was used to determine the elemental composition of the various catalysts. Samples were prepared by dissolving the catalyst powders in a mixture of H_2SO_4, HNO_3 and HCl (1:1:3). The solutions were heated from room temperature to 180 °C in 10 min using a Microwave Discover SP -D (CEM Corporation) and maintained at this

temperature for 20 min. Finally, the solutions were diluted with Milli-Q® water, filtered and brought to a known volume. To calculate the concentration of the different solutions, 5 standards were prepared for Pt, Ni and Mo with concentrations of 0, 1, 4, 7 and 12 mg/L of each element.

On-line ICP-MS measurements

Stability of the oh-PtNi and the Rh-, and Mo-doped PtNi electrocatalyst samples was studied with on-line ICP-MS. The setup consists of a custom-designed and manufactured propylene carbonate scanning flow-cell (SFC). The outlet of the cell was coupled to the inlet of the ICP-MS (Perkin Elmer NexION 350X). A GC rod was used as a counter electrode that was connected to the SFC via a T-connector on the inlet side. An Ag/AgCl/ 3 M KCl reference electrode (Metrohm) was connected to the cell via a capillary channel on the outlet side. All potentials in the manuscript are reported against the reversible hydrogen electrode (RHE). Electrocatalyst spots were drop-casted on a GC plate (SIGRADUR, A = 25 cm^2), which was used as the working electrode. Inks were prepared according to the recipe described below in the section "Electrochemical measurements." The only difference is that only 0.2 µl (instead of 10 µl) of catalyst suspension was drop-casted onto the polished glassy carbon plate resulting in spot diameters between 1200–1500 µm. The working electrode was sitting on an XYZ translation stage (Physik Instrumente M-403), allowing rapid navigation in between samples. Electrochemical protocols were performed using a Gamry Reference 600 potentiostat. An in-house developed LabVIEW software controlled all instruments, including the stages, gas control box, and the potentiostat. ICP-MS was calibrated daily by a four-point calibration slope prepared from standard solutions (Pt, Ni, Mo, Rh, Re, Co, In – Merck Centripur). ^{187}Re, ^{58}Co, ^{115}In were used as internal standards. The sample and internal standard streams were merged via a Y-connector right before the nebulizer of the ICP-MS. The Ar-purged electrolyte flow was controlled by the peristaltic pump of the ICP-MS (Elemental Scientific, M2 pump) with an average flow-rate of 3.48 µl s^{-1}. Electrochemical measurements were performed in 0.1 M $HClO_4$ electrolyte solution saturated with Ar. Contact with the SFC was established at OCP. The system was held at this potential for 5 min, followed by 10,800 cycles between 0.6–0.95 V_{RHE} applying 1 V s^{-1} scan rate. The protocol was finished with performing one CV in the potential window of 0.6–1.5 V_{RHE} applying 10 mV s^{-1} scan rate and a potentiostatic hold at 0.6 V_{RHE} for 5 min (allowing the ICP-MS signal to return to its baseline). The composition of the electrocatalysts after the AST protocol was calculated using the composition and Pt mass loading determined by ICP-OES measurements. Only mass loss before the final CV was considered.

Synchrotron Wide-Angle X- Ray Scattering (WAXS) Measurements.

WAXS measurements were performed at ID31 beamline of the European Synchrotron Radiation Facility (ESRF) in Grenoble, France. The samples were measured in grazing-incident geometry in a customized three-electrode-operando-cell connected to a BioLogic SP-300 potentiostat. Catalyst films were prepared via drop-casting of ink on a mirror-polished glassy carbon cylinder that was connected as working electrode from the cylinders bottom to the cell. A Pt wire acted as counter electrode and a leak less miniature Ag/AgCl electrode as reference electrode (ET072, eDAQ). The electrolyte (0.1 M $HClO_4$, Suprapur®, Merck) was continuously pumped through the cell at a flow rate of 20 mL·min^{-1} using a peristaltic pump. The high-energy X-ray beam (78 keV) was focused on the sample and the scattered signal collected using a Dectris Pilatus CdTe 2M detector positioned 850 mm behind the sample. The energy, detector distance and tilts were calibrated using a standard CeO_2 powder and the 2D diffraction patterns were reduced to the presented 1D curves using the pyFAI software package [Ashiotis, G.; Deschildre, A.; Nawaz, Z.; Wright, J. P.; Karkoulis, D.; Piccac, F. E.; Kieffera, J., The fast azimuthal integration Python library: PyFAI. *J. Appl. Crystallogr.* 2015, *48*, 510-519.].

Rietveld Refinements.

Rietveld refinement of the WAXS patterns was performed to extract the phase structure, crystallite size and lattice parameter using the Fm3m structure of Pt metal (with possibly Ni oxide) and the Fullprof software. The instrumental resolution function was determined by the refinement of a CeO2 standard sample. Thomson-Cox-Hastings profile function was adopted. The background of patterns was described by an interpolated set of points with refinable intensities. When the contribution of the glassy carbon electrode substrate was visible on the diffraction patterns, the Rietveld analysis was restricted to the preserved Pt [220] Bragg reflection.

Electrochemical Measurements.

A typical three-electrode cell was used to perform the electrochemical measurements. A rotating disk electrode (RDE) made of glassy carbon (0.196 cm^2, Pine Instrument) served as the working electrode. A hydrogen reference electrode (Gaskatel, HydroFlex) and a Pt mesh attached to a Pt wire were used as reference electrode and counter electrode, respectively. A catalyst ink was prepared by mixing 5.5 mg of the as-prepared catalyst powders (PtNi/C and PtNi(RhMo)/C) in water, isopropanol, Nafion (v/v/v= 3.98 ml / 1.00 ml / 0.01 ml), totaling 4.99 ml, and sonicated for 45 min using an ultrasonic horn sonicator in an ice bath. 10 µL of the catalyst ink were dropped onto the working electrode and dried at 60 ℃ for 7 min. The loading amount of the metal Pt for the octahedral PtNi and PtNi(RhMo)/C catalyst was 1.3-1.5 μg_{pt}. For comparison, the commercial Pt/C catalyst JM was used as benchmark catalysts with a Pt loading of 10 $\mu g_{Pt}/cm^2$. Cyclic voltammetry (CV) measurements were performed in a 0.1 M $HClO_4$ nitrogen-saturated solution, with first 50 cycles of CVs at a scan rate of 100 mV s^{-1} for activation and then cycles at a rate of 20 mV s^{-1} for ECSA acquisition. Oxygen reduction response (ORR) measurements were performed in a 0.1 M $HClO_4$ solution that was purged with oxygen during the measurement. Linear sweep voltammetry (LSV) was performed at a scan rate of 20 mV s^{-1} and ORR polarization curves were recorded at 1600 rpm. We note that the scan rate has an impact on the mass-normalized current, i.e., higher scan rate usually reports higher performance. In our earlier benchmarking paper,[1] we addressed this issue with reference to the literature. In general, scan rates in the range of 10-20 mV s^{-1} are recommended because they are not too high, so that capacitive currents are minimized and possible overcorrection is avoided, and also not too low, because in this case the amount of OH_{ads} on the surface and adsorption of impurities could be large and reduce activity. For analysis of the ORR activity, LSV curves in oxygen were corrected by LSV curves in nitogen. The ECSAs were determined by integrating the hydrogen adsorption charge on the CV and from CO-stripping charges.

For CO-dossing experiments, CO was adsorbed on the electrode by keeping the potential at 0.05 V_{RHE} (dossing potential), for 60 s, after which CO was adsorbed in CO-saturated solution for 25 s, followed by 10 min of N_2 flushing. Followed by CVs to CO-stripping

and CV after CO oxidation from 0.05 to 1 V_{RHE} and back to 0.05 V_{RHE}. Measurements were performed in a 0.1 M $HClO_4$ N_2-saturated solution at a potential scan rate of 50 mV s^{-1}.

Results and Discussion

Table S1. ICP-OES metallic at%, Pt wt% of octahedral PtNi(L)/C samples, and their corresponding mean edge length distribution in nm for the as-prepared particles.

Sample	ICP-OES		Mean edge length
	Metallic at%	Pt wt%	nm
PtNi	$Pt_{18}Ni_{82}$ /C	11.7	6.5 ± 0.6
PtNi(RhMo)	$Pt_{32.6}Ni_{65.6}Rh_{1.4}Mo_{0.4}$/C	14.65	8.8 ± 0.6

*IL-STEM-EDS elemental measurements were repeated on several single particles from the same batch

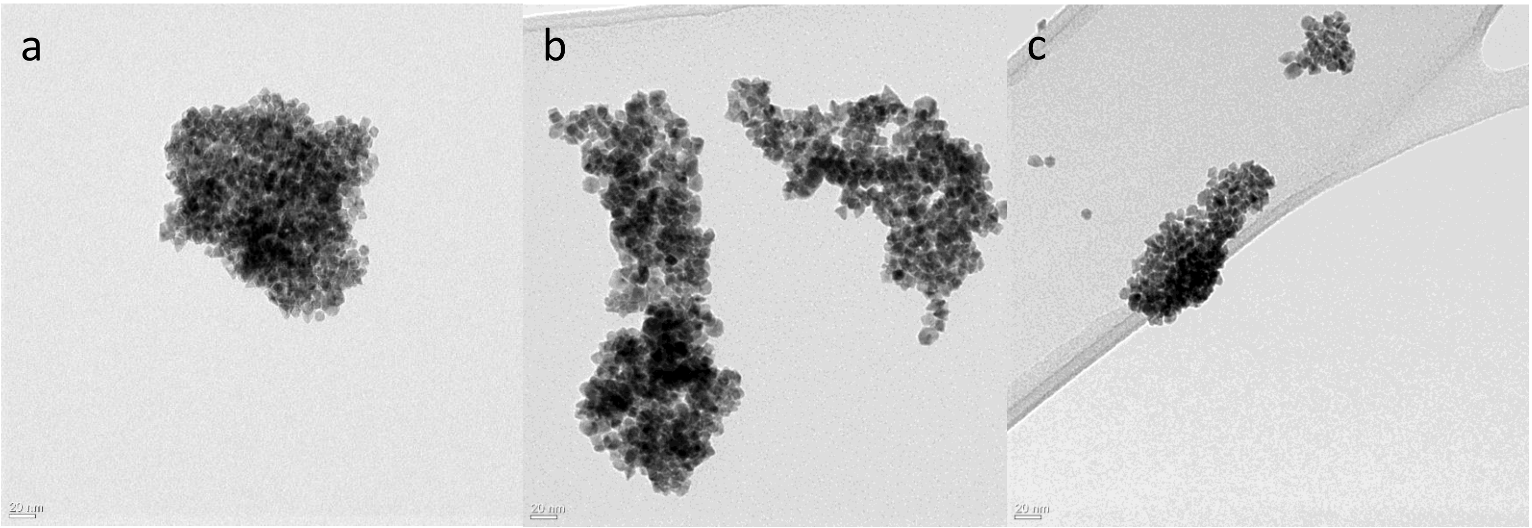

Figure S1. TEM images of PtNi(Rh) particles produced with different Rh precursors a) $Rh(acac)_3$ 40 mg, b) $Rh(acac)_3$ 20 mg and c) $Rh(acac)(CO)_2$ 13 mg

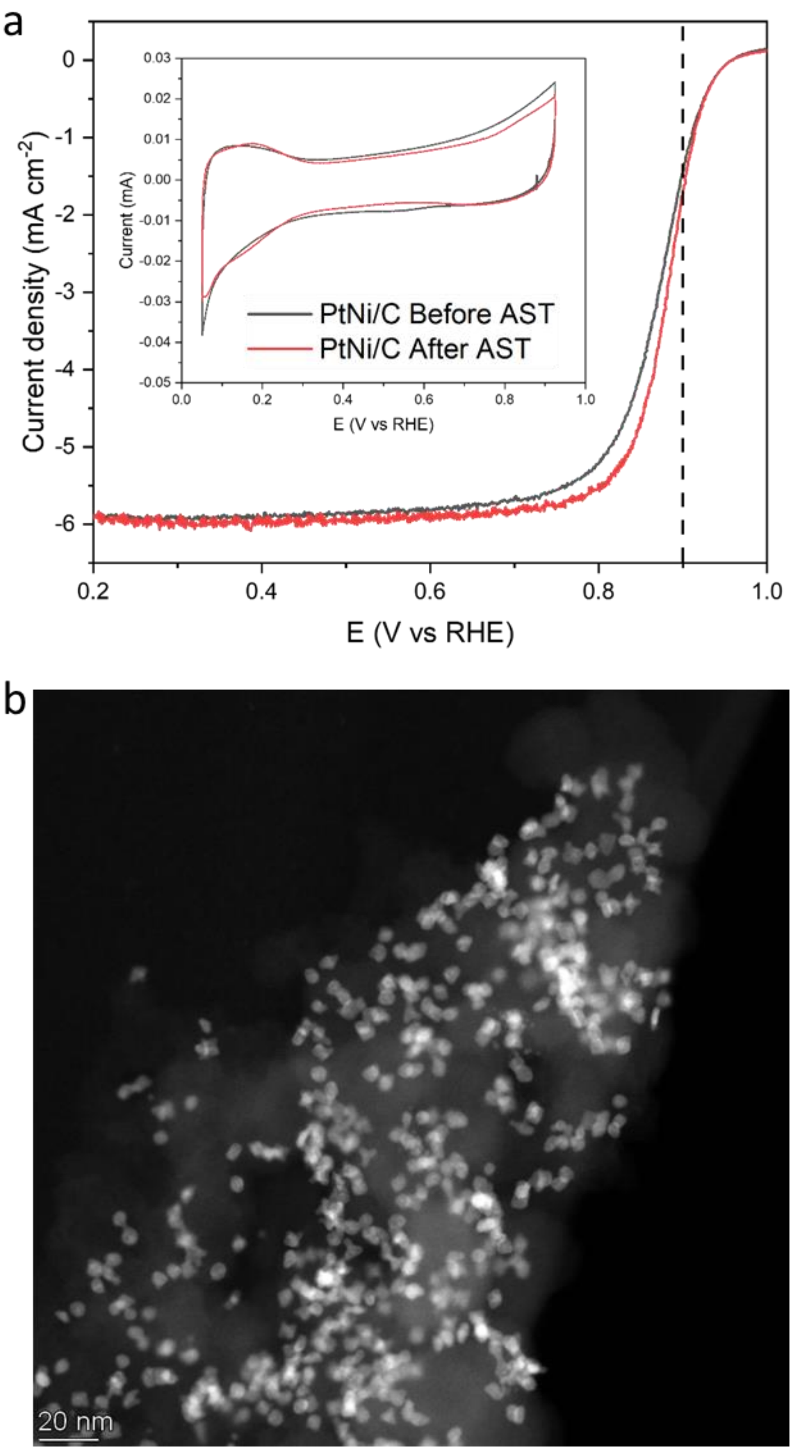

Figure S2. Electrochemical characterization of oh-PtNi. a) Linear sweep voltammetry curves before and after the stability test. Inset shows the cyclic voltammetry profile before and after the stability test. b) TEM image of oh-PtNi catalysts after the stability test as probed directly from the RDE working electrode.

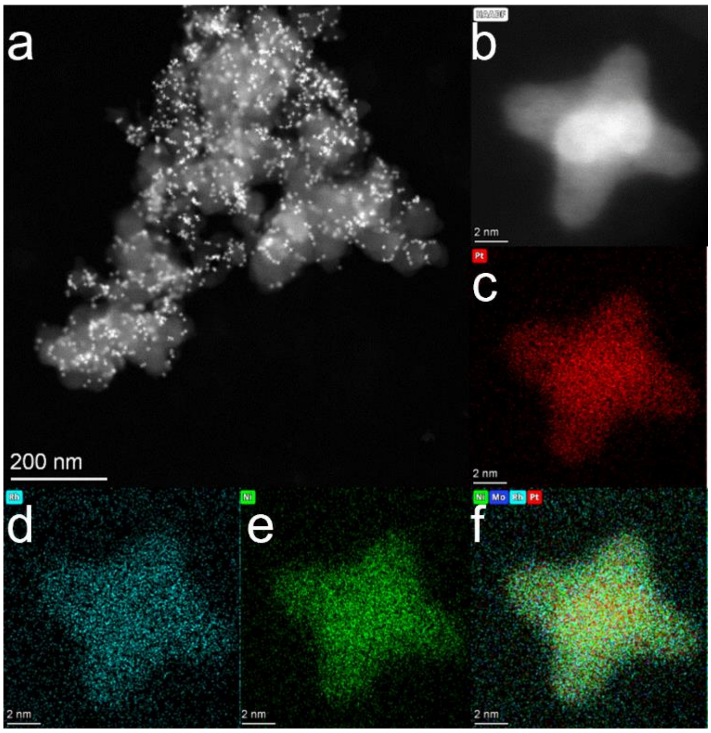

Figure S3. Morphological and compositional characterization of oh-PtNi(RhMo) after the stability test as probed directly from the RDE working electrode. a,b) HAADF-STEM micrograph. c-f) STEM-EDX elemental composition maps.

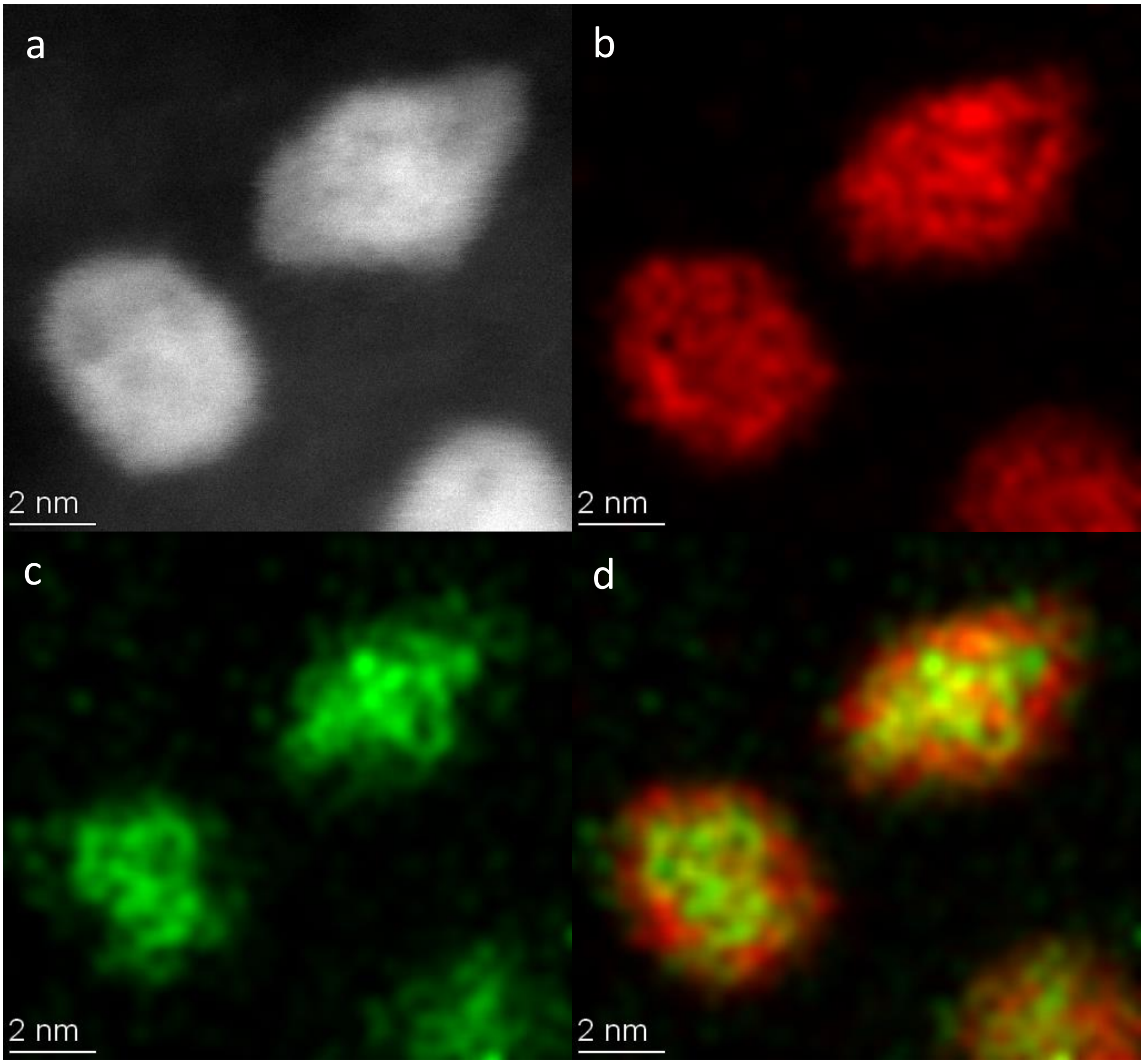

Figure S4. Morphological and compositional characterization of binary oh-PtNi after the stability test as probed directly from the RDE working electrode. a) HAADF-STEM micrograph. b-f) STEM-EDX elemental composition maps (Pt, red and Ni, green).

Table S2. Electrochemical performance and stability. $ECSA_{Hupd}$ and $ECSA_{CO}$ before and after ASTs, mass and specific activities before and after ASTs (evaluated at 0.9 V_{RHE}).

Catalysts	ECSA<br>(m^2/g_{Pt})		Specific Activity<br>@ 0.9 V<br>(mA/cm^2)		ECSA<br>(m^2/g_{Pt})		Specific Activity<br>@ 0.9 V<br>(mA/cm^2)		Mass Activity<br>@ 0.9 V<br>(A/mg_{Pt})		AST<br>Cycles<br>(sweeping rate)<br>Potential<br>window V_{RHE}
	Based on H_{upd}				*Based on CO-stripping*						
	Before<br>AST	After<br>AST	Before<br>AST	After<br>AST	Before<br>AST	After<br>AST	Before<br>AST	After<br>AST	Before<br>AST	After AST	
Pt/C	67.7	-	0.46	-	-	-	-	-	0.31	-	
oh-Pt<br>Hornberger et al.[2]	36	32	1.10	0.94	34				0.36	0.31	10,000<br>(100 mV/S)<br>0.6–0.925
oh- $Pt_{18}Ni_{82}$[a]	33.5	37.1	0.50	0.61	65.9	55.0	0.48	0.73	0.32	0.40	10,800<br>(1 V/S)<br>0.6–0.95
oh-$Pt_{40}Ni_{60}$<br>Cui et al.[3]			1.97	2.89					0.68	1.02	25[b]<br>(250 mV/S)<br>0.06–1.0
oh-$Pt_{33}Ni_{67}$<br>Beermann et al.[4]	33.4	46.5			28.3	43.8			1.7	1.44	4,000<br>(50 mV/S)<br>0.5–1.0
oh-$Pt_{71}Ni_{26}(Rh_3)$<br>Beermann et al.[5]	30.2	33 (4k)<br>33.1 (8k)	2.65	3.45(4k)<br>2.16(8k)<br>1.17(30k)					0.82	1.14(4k)<br>0.72(8k)<br>0.32(30k)	30,000<br>(50 mV/S)<br>0.5–1.0
oh-$Pt_{66.6}Ni_{31.9}(Mo_{1.5})$<br>Dionigi et al.[6]	38	44.7	9.04	-	51.7	-	6.86	-	3.43	2.46	10,000<br>(100 mV/S)<br>0.6–0.925
oh-$Pt_{73.9}Ni_{24.5}(Mo_{1.6})$<br>Huang et al.[7]	67.7	-	10.3	9.7	83.9	-	8.2	-	6.98	6.6	8,000<br>(50 mV/S)<br>0.6–1.1
oh-$Pt_{27}Ni_{71.4}(Mo_{1.6})$<br>Polani et al.[8]	55.0	60.7	4.13	2.94	61.7	55.7	3.68	2.90	2.27	1.61	10,800<br>(1 V/S)<br>0.6–0.95
oh-$Pt_{32.6}Ni_{65.6}(Rh_{1.4}Mo_{0.4})$[a]	52.6 ±<br>10.1	62 6 ±<br>4.0	2.93 ±<br>0.11	3.38 ±<br>0.12	54.8 ± 6.7	56.4 ±<br>2.5	2.55 ±<br>0.06	3.75 ±<br>0.13	1.53 ±<br>0.25	2.11 ± 0.14	10,800<br>(1 V/S)<br>0.6–0.95

[a] This work

[b] Activation

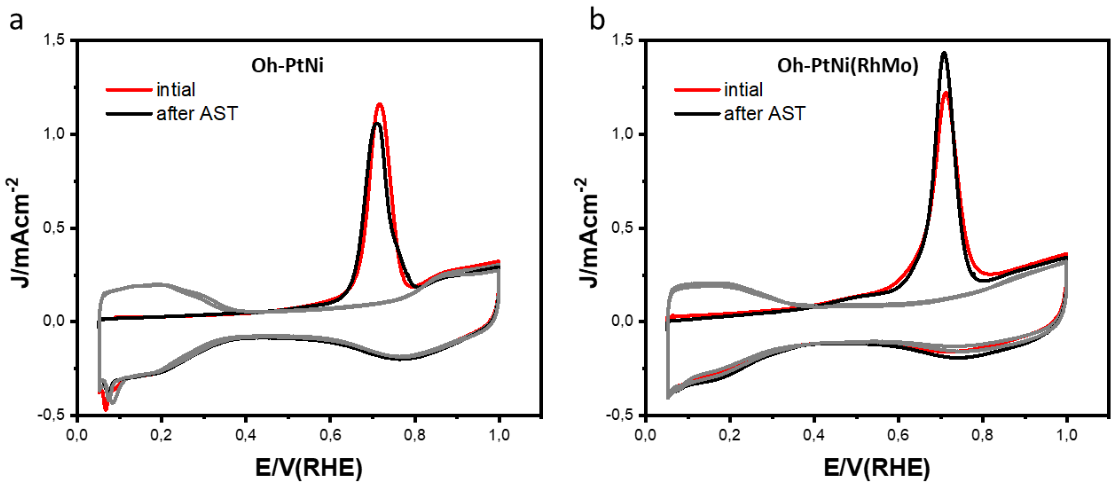

Figure S5. CO-Stripping curves before and after ASTs for (a) oh-PtNi and (b) oh-PtNi(RhMo) catalysts.

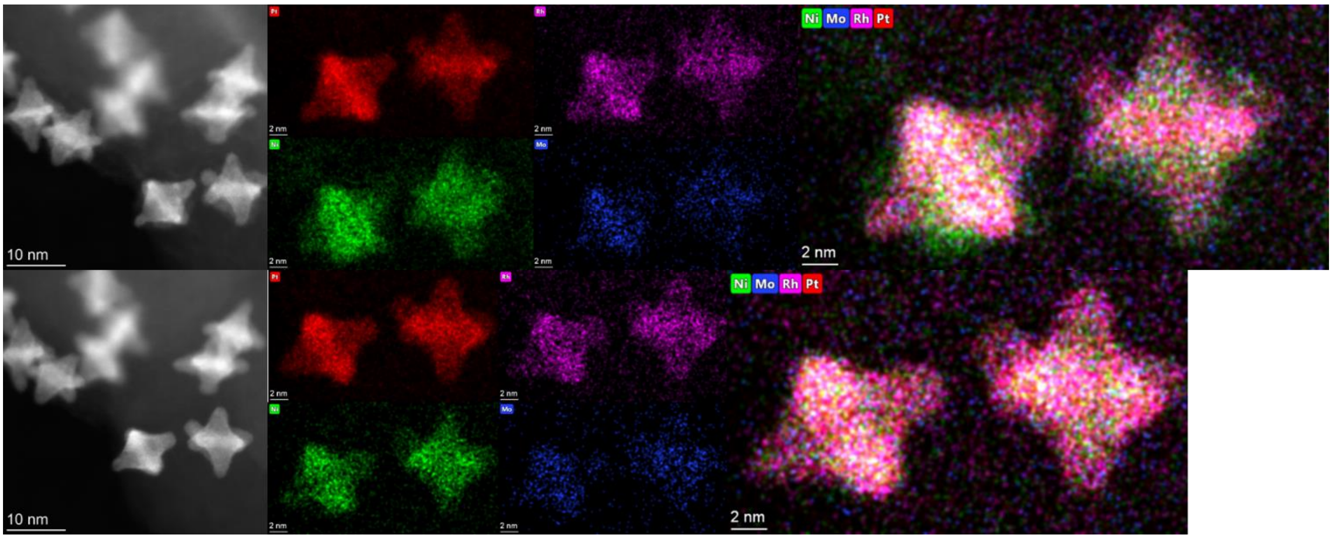

Figure S6. IL-HAADF-STEM-EDX for oh-PtNi(RhMo) catalyst before (top) and after (bottom) AST.

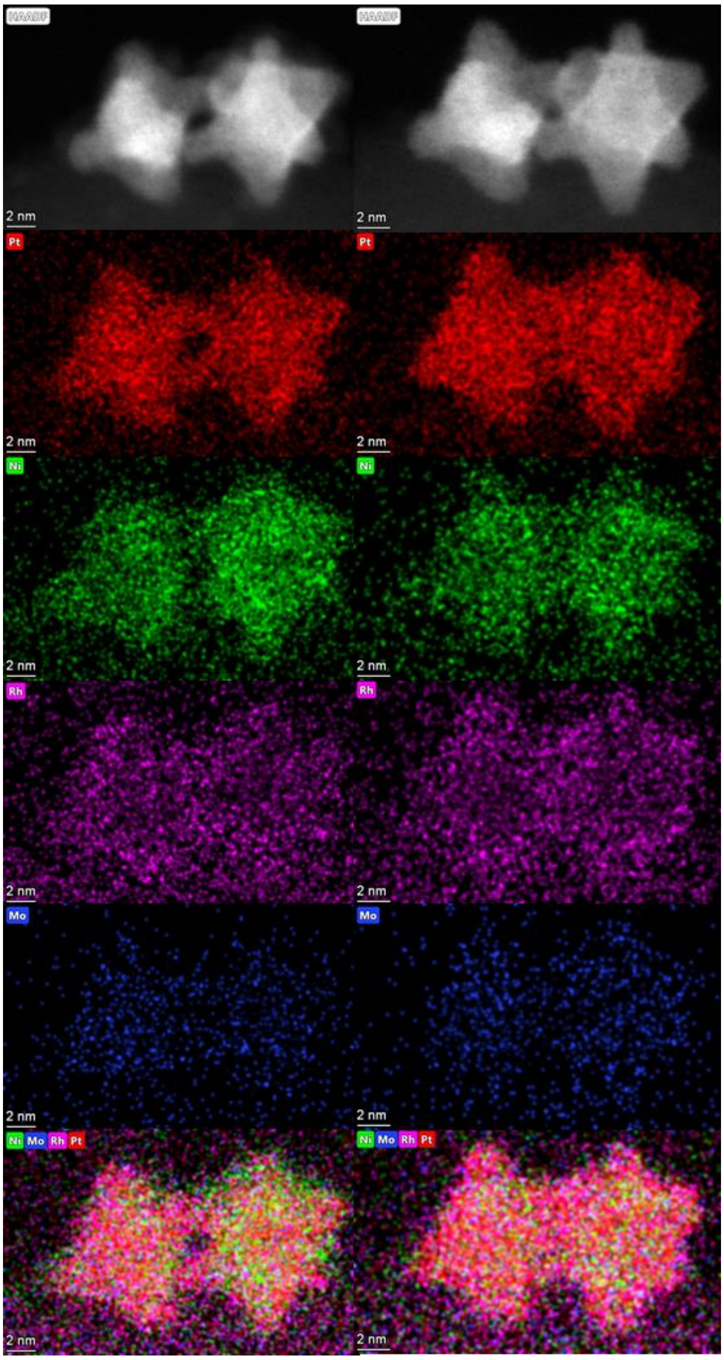

Figure S7. IL-HAADF-STEM-EDX for oh-PtNi(RhMo) catalyst before (left) and after (right) AST.

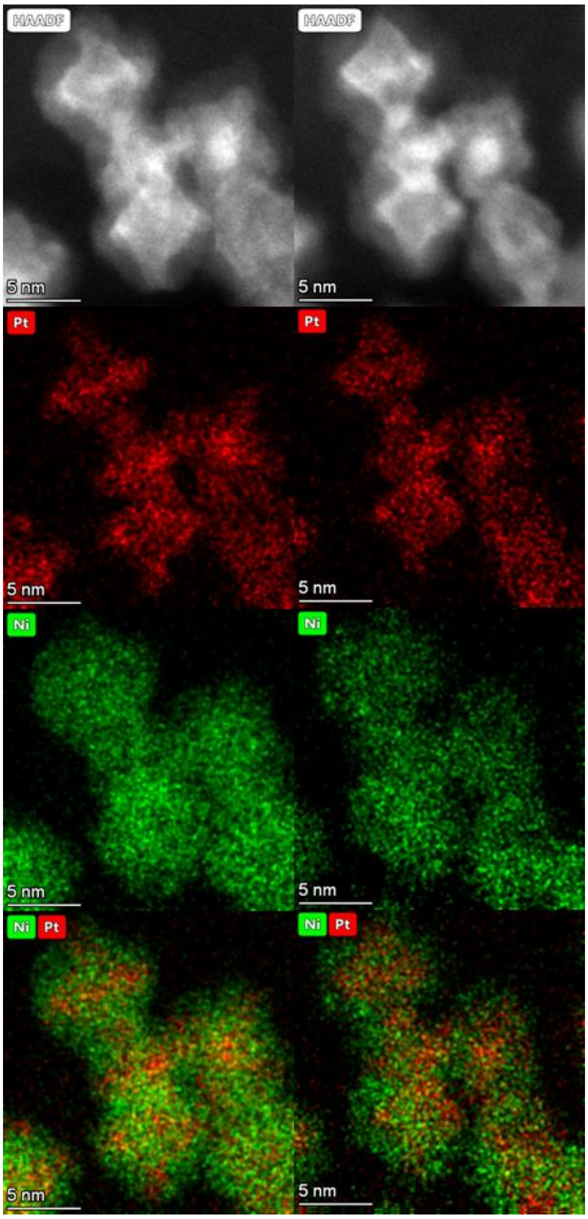

Figure S8. IL-HAADF-STEM-EDX for oh-PtNi catalyst before (left) and after (right) AST.

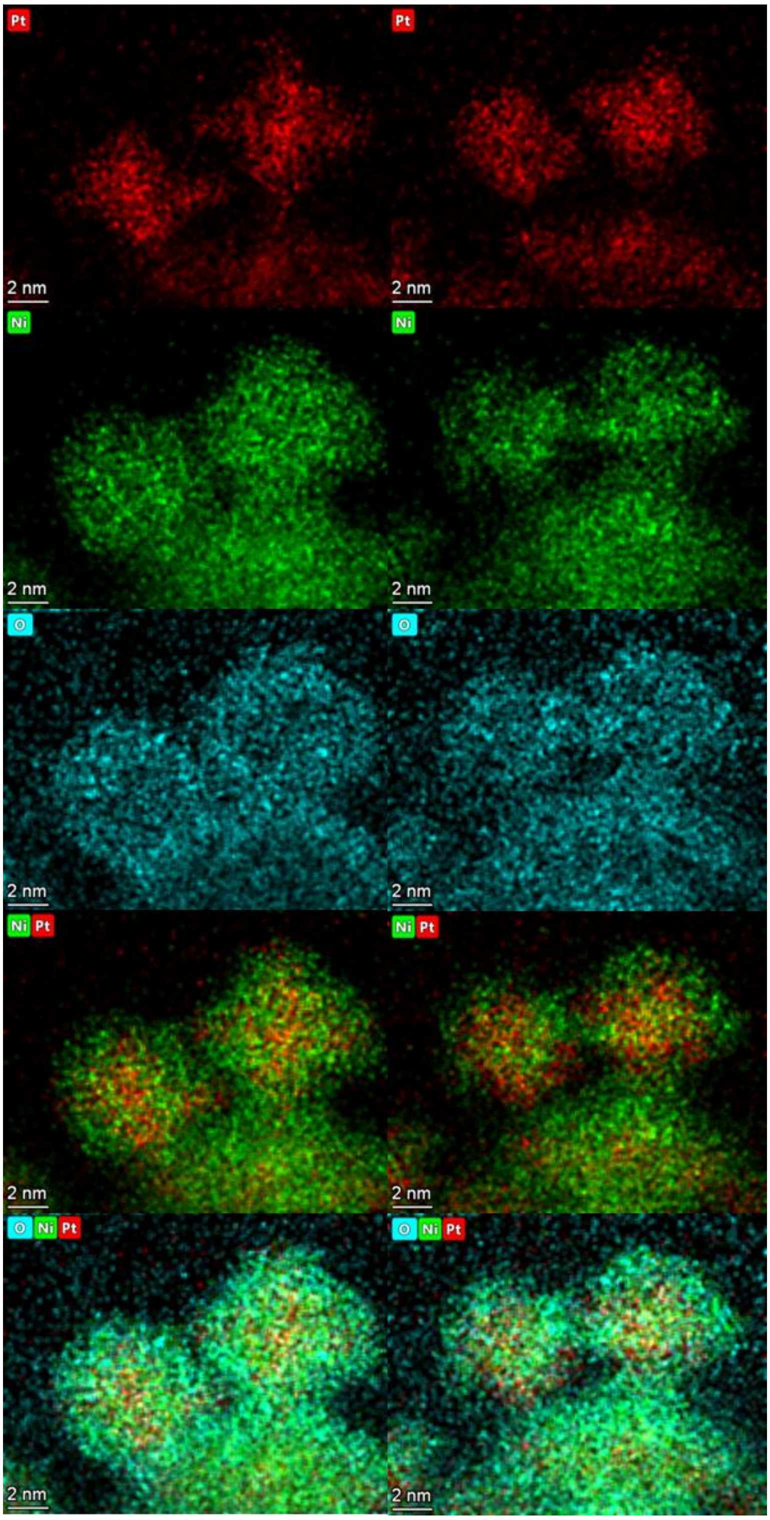

Figure S9. IL-STEM-EDX for oh-PtNi catalyst before (left) and after (right) AST showing the O Ka elemental maps.

Table S3. IL-STEM-EDX and online SFC-ICP-MS metallic at% of octahedral samples before and after AST.

Sample	IL-STEM-EDX metallic at% *(replications)		On-line SFC-ICP-MS	
	Before AST	After AST	Before AST (based on ICP-OES)	After AST
oh-PtNi	$Pt_{14.4}Ni_{31.3}O_{54.3}$ (5)	$Pt_{15.2}Ni_{23.4}O_{61.4}$ (5)	$Pt_{18}Ni_{82}$	$Pt_{21}Ni_{79}$
oh-PtNi(RhMo)	$Pt_{42.2}Ni_{48.6}Rh_{2.8}Mo_{6.4}$ (5)	$Pt_{59}Ni_{28}Rh_{5.4}Mo_{7.6}$ (9)	$Pt_{32.6}Ni_{65.6}Rh_{1.4}Mo_{0.4}$	$Pt_{43.88}Ni_{54.16}Rh_{1.78}Mo_{0.18}$

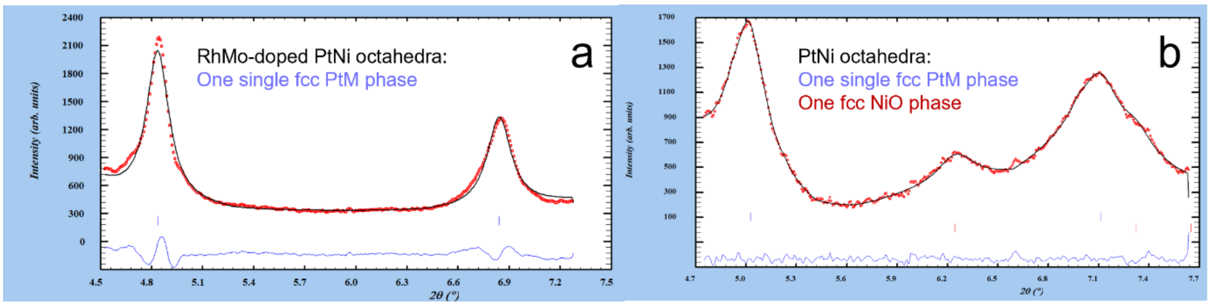

Figure S10. Rietveld refinement fits (red) of the background subtracted WAXS partial pattern (black) using for one single fcc PtM phase (purple) for a) RhMo-doped PtNi octahedra and one single fcc PtM phase (purple) and one fcc NiO phase (dark red) for b) PtNi octahedra and the difference curve (blue).

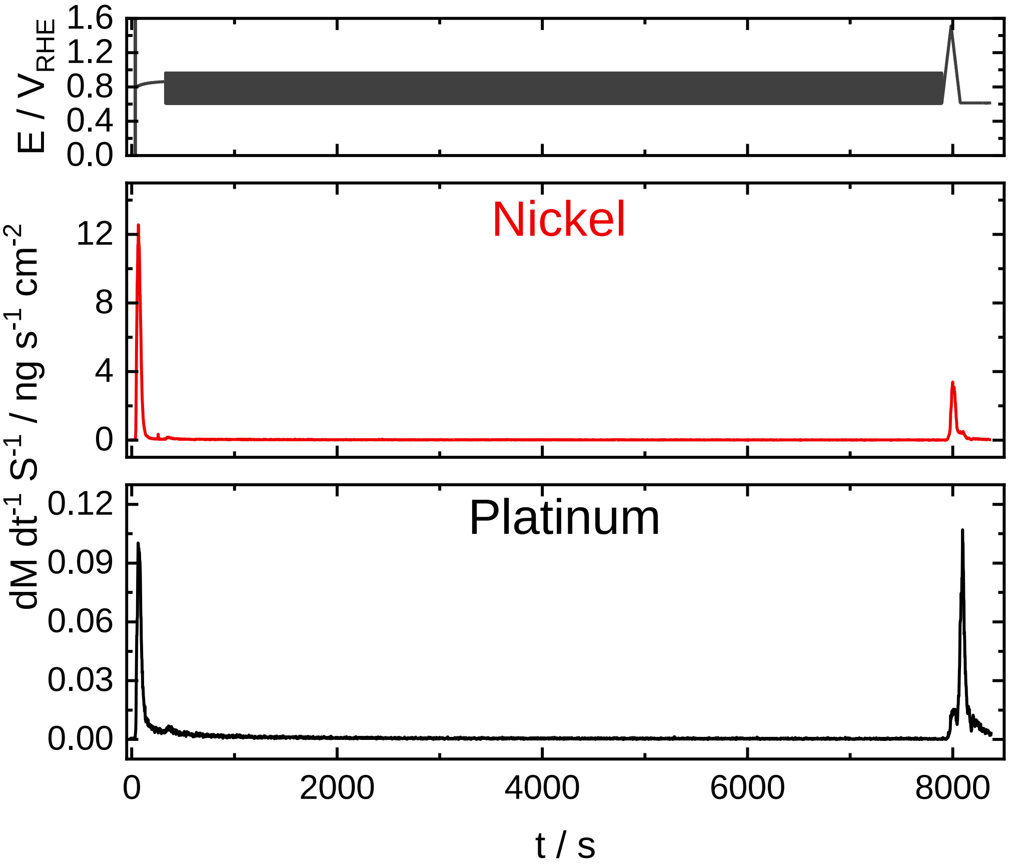

Figure S11. Dissolution profiles recorded for the oh-PtNi catalyst. Electrochemical measurements were performed in 0.1 M $HClO_4$ electrolyte solution saturated with Ar. Contact with the SFC was established at OCP. The system was held at this potential for 5 min, followed by 10,800 cycles between 0.6–0.95 V_{RHE} applying 1 V s^{-1} scan rate. The protocol was finished with performing one CV in the potential window of 0.6–1.5 V_{RHE} applying 10 mV s^{-1} scan rate and a potentiostatic hold at 0.6 V_{RHE} for 5 min (allowing the ICP-MS signal to return to its baseline).

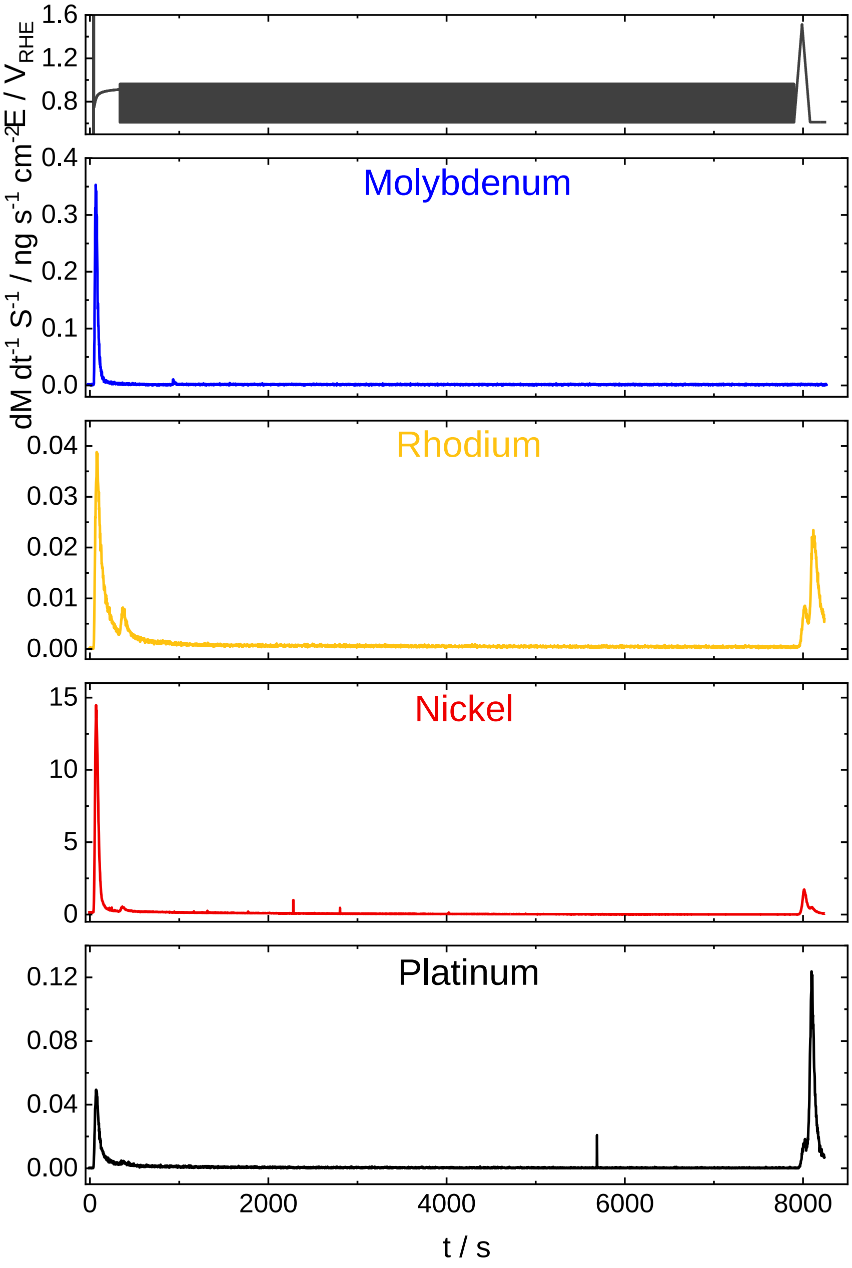

Figure S12. Dissolution profiles recorded for the oh-PtNi(RhMo) catalyst. Electrochemical measurements were performed in 0.1 M $HClO_4$ electrolyte solution saturated with Ar. Contact with the SFC was established at OCP. The system was held at this potential for 5 min, followed by 10,800 cycles between 0.6–0.95 V_{RHE} applying 1 V s^{-1} scan rate. The protocol was finished with performing one CV in the potential window of 0.6–1.5 V_{RHE} applying 10 mV s^{-1} scan rate and a potentiostatic hold at 0.6 V_{RHE} for 5 min (allowing the ICP-MS signal to return to its baseline).

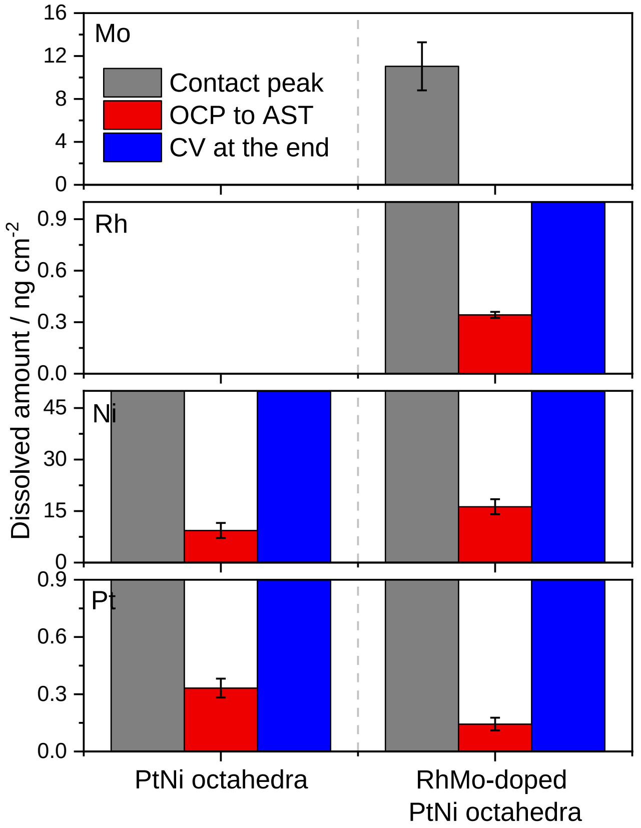

Figure S13. Magnified sections of Figure 5.

References

1. Martens, S. *et al.* A comparison of rotating disc electrode, floating electrode technique and membrane electrode assembly measurements for catalyst testing. *J. Power Sources* **392**, 274–284 (2018).
2. Hornberger, E. *et al.* Seed-Mediated Synthesis and Catalytic ORR Reactivity of Facet-Stable, Monodisperse Platinum Nano-Octahedra. *ACS Appl. Energy Mater.* **4**, 9542–9552 (2021).
3. Cui, C., Gan, L., Heggen, M., Rudi, S. & Strasser, P. Compositional segregation in shaped Pt alloy nanoparticles and their structural behaviour during electrocatalysis. *Nat. Mater.* **12**, 1–7 (2013).
4. Beermann, V. *et al.* Tuning the Electrocatalytic Oxygen Reduction Reaction Activity and Stability of Shape-Controlled Pt-Ni Nanoparticles by Thermal Annealing -Elucidating the Surface Atomic Structural and Compositional Changes. *J. Am. Chem. Soc.* **139**, 16536–16547 (2017).
5. Beermann, V. *et al.* Rh-Doped Pt-Ni Octahedral Nanoparticles: Understanding the Correlation between Elemental Distribution, Oxygen Reduction Reaction, and Shape Stability. *Nano Lett.* **16**, 1719–1725 (2016).
6. Dionigi, F. *et al.* Controlling Near-Surface Ni Composition in Octahedral PtNi(Mo) Nanoparticles by Mo Doping for a Highly Active Oxygen Reduction Reaction Catalyst. *Nano Lett.* **19**, 6876–6885 (2019).
7. Huang, X. *et al.* High-performance transition metal – doped Pt 3 Ni octahedra for oxygen reduction reactionHuang, X., Cao, L., Chen, Y., Zhu, E., Lin, Z., Li, M., … Mueller, T. (2015). High-performance transition metal – doped Pt 3 Ni octahedra for oxygen reduction reactio. *Science (80-.).* **348**, 1230–1234 (2015).
8. Polani, S. *et al.* Size and Composition Dependence of Oxygen Reduction Reaction Catalytic Activities of Mo-Doped PtNi/C Octahedral Nanocrystals. *ACS Catal.* 11407–11415 (2021) doi:10.1021/acscatal.1c01761.

Author Contributions

E.H., M.K. and S. P. analyzed the data and composed the manuscript. S. P. synthesized the catalysts. L. P. and E. H. performed RDE experiments. M. K. and X. W. performed TEM experiments. P. P. and K. M. performed and analyzed the IL-STEM-EDX experiment. R. C. and J. D. performed and analyzed the WAXS experiment. A.K. and S.C. performed and analyzed the SFC-ICP-MS experiment. P. S., R. D.-B. and M. H. supervised the study.
